# High-density “APLE” Mapping—Activation, Propagation, Low Voltage, and Electrogram Evaluation with the HD Grid for Atrioventricular Nodal Re-entry Tachycardia Ablation

**DOI:** 10.19102/icrm.2024.15034

**Published:** 2024-03-15

**Authors:** Nitin Somasundaram, Nicholas H. Von Bergen

**Affiliations:** 1 The Medical College of Wisconsin, Milwaukee, WI, USA; 2 Department of Pediatrics, The University of Wisconsin School of Medicine and Public Health, Madison, WI, USA

**Keywords:** Ablation, atrioventricular nodal re-entry tachycardia, cryotherapy, slow pathway, voltage mapping

## Abstract

This is the first case series to evaluate high-density mapping of the triangle of Koch (TOK) using the HD Grid to guide slow-pathway ablation integrating activation, propagation (with wave collision), low-voltage signals, and atrial electrogram appearance. We will describe our technique and the results in this case series. Using three-dimensional mapping and the HD Grid, patients underwent high-density voltage mapping of the TOK. Ablation site selection was based on properties during sinus rhythm with late activation, at or above the propagation wave collision, over low voltage, and with appropriate electrogram appearance. Five patients underwent mapping of the slow pathway using the HD Grid. Their median age was 14 years, their median weight was 54.1 kg, and their median height was 161.5 cm. The TOK was mapped with the HD Grid for a median of 3 min. The procedure was successful in all patients using this technique. The median lesion number to the site of success was 3, with a median total number of cryotherapy lesions of 11. No radiation was used. There were no recurrences. Using activation, propagation wave, low voltage, and electrogram appearance when mapping for slow-pathway localization and ablation with the HD Grid can be successful, results in high-density maps, and is relatively faster.

## Introduction

Atrioventricular (AV) nodal re-entrant tachycardia (AVNRT) remains the most common supraventricular tachycardia (SVT) until the onset of atrial fibrillation in older age.^1,2^ Ablation of the slow pathway for the treatment of AVNRT provides a long-term cure in the great majority of cases. Even so, despite decades of experience with slow-pathway ablation, the ideal mapping technique remains to be defined. An anatomic approach, using landmarks for the triangle of Koch (TOK) along with the appearance of the atrial electrogram, is commonly adopted. Unfortunately, frequent recurrences and a small but real risk of AV nodal injury remain.^3^ In an attempt to improve the accuracy of slow-pathway localization and ablation, voltage mapping and electrically guided techniques have been used to assist with slow-pathway site selection.^4,5^ Even so, voltage mapping alone demonstrates low sensitivity and specificity.^6^ A desire for improvements in accuracy for slow-pathway ablation has prompted the search for other electrical markers to guide slow-pathway ablation, including integrating the timing of the atrial signals during sinus rhythm.^7^

As voltage mapping and activation mapping require acquisition of the atrial signals within the TOK, high-density point collection could potentially improve the accuracy of these techniques. Mapping has been trialed using the HD Grid to map low-voltage areas^8^; however, this is the first study to evaluate the HD Grid to guide slow-pathway ablation using activation, propagation (with wave collision), low-voltage signals, and atrial electrogram interpretation (known as the APLE mapping technique). Here, we will describe the use of the APLE mapping technique and its results in a small case series.

## Case series

Five patients underwent AVNRT ablation using the HD Grid procedure including the APLE technique described later. Patients were <18 years of age at the time of the procedure. Patient demographics are described later. All patients had documented SVT prior to their procedure.

As this was a retrospective evaluation, the need for informed consent was waived for this study. This study was approved by the University of Wisconsin Institutional Review Board.

## Methods

### Description of the procedure

#### Three-dimensional geometry creation and atrial electrogram collection

Patients presented to the cardiac catheterization lab in a fasting state. They were induced and maintained under general anesthesia. All procedures were performed radiation-free using the EnSite Precision™ Navigation System (Abbott, Chicago, IL, USA). A steerable decapolar 5-French (Fr) catheter (IBI Medical, Irvine, CA, USA) was advanced to the heart, and right atrial geometry was collected. The His signal was labeled on the three-dimensional (3D) map before placement of the catheter in the coronary sinus (CS) for pacing and recording. A second 5-Fr decapolar catheter, which had electrodes for both the His bundle and the right ventricular apex (IBI Medical), was placed in the appropriate position past the His bundle to the right ventricle using the premade 3D map. A baseline study was completed off isoproterenol, evaluating for markers of AVNRT or the slow pathway. If there was no inducible AVNRT, then isoproterenol was started (0.02 μg/kg/min) to assist with induction. If SVT was induced, then AVNRT was confirmed with ventricular overdrive pacing before proceeding to mapping and ablation.

After AVNRT confirmation, a (donated) HD Grid was placed through an 8-Fr sheath, and heparin was given. The HD Grid was used to map the TOK during sinus rhythm. Once an adequate atrial signal point density was obtained at and around the TOK, as determined by the electrophysiologist, attention was directed toward evaluating the mapped signals for ablation site selection using the APLE technique. Any point not considered directly on the surface of the 3D map within the TOK was not included in the subsequent analysis.

#### Using activation, propagation, low voltage, and the electrogram for ablation site selection

This technique, termed the APLE technique, evaluates the local activation time, the propagation map for a wave collision, low-voltage signals, and atrial electrogram. The site selected for ablation is in an area of later activation (local activation time [LAT] map), at a site with a low-voltage signal (voltage map), at or superior to the wave collision (propagation map), and at a site with a long-duration atrial electrogram.

#### Activation mapping and propagation wave mapping

**Figure 1:**
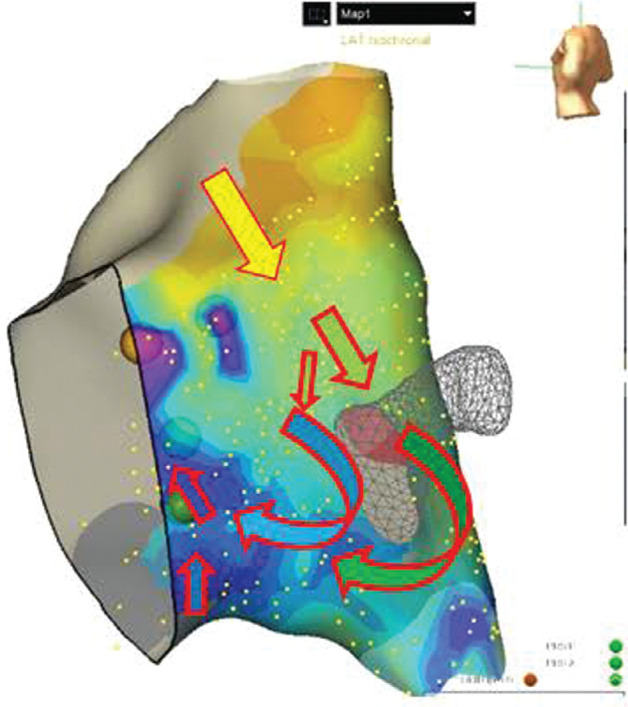
An example of a local activation map for patient 1 demonstrating the direction of atrial signal conduction during sinus rhythm when viewing the right atrium from a virtual left posterior lateral view. Orange denotes the earliest and purple denotes the latest local activation time, respectively. The red arrows (added by the authors) represent the direction of the atrial signal movement. Areas of late activation, distant from the His, after the change in direction are considered as potential sites of ablation after considering other parts of the APLE technique. *Abbreviations:* APLE, activation, propagation, low-voltage, and electrogram; LAT, local activation time.

**Figure 2:**
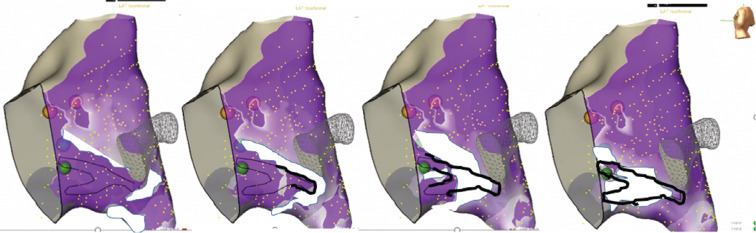
The white color represents the atrial signal activation for patient 1 (edited by the authors to highlight the advancing wavefront). Each image is a slice in time with later images to the right. The site of propagation waves colliding in the TOK where the sinus rhythm waves merge is marked just surrounding the site where the two wavefronts collide. The thin dark line mapped at the time of the procedure (left image) is emphasized by the authors in the additional images to highlight the process of wave collision mapping.

The LAT, measured at last deflection on the atrial signal, and the propagation map were used to evaluate for areas of late activation and assess the location where the atrial signals converge, termed the wave collision **(Figures 1 and 2)**. **Figure 1** is an LAT map with arrows overlying the map (added by the authors) to demonstrate the direction of electrical flow during sinus rhythm. The sinus rhythm activation of the TOK typically conducts more rapidly on the posterior/atrial side of the tendon of Todaro and then progresses both superiorly and inferiorly around the CS ostium to enter the TOK. The propagation wave then typically alters the direction (a pivot point) from the more inferior direction to rotate toward the ventricle and then superiorly toward the His bundle. Areas of later activation, typically after the pivot point, are selected as sites to consider for ablation. A sparkle map can also be used to evaluate the duration of atrial signals, though it was not used for site selection in our patients. Please see **Videos 1 and 2** for examples of the propagation wave and sparkle map.

When the atrial signals are evaluated as a propagation wave **(Figure 2)**, the slower wavefront progressing from the superior TOK collides with the more progressive wavefront entering from the low TOK. This site, termed the site of propagation wave collision, is marked on the map. Potential sites for slow-pathway ablation were considered at or superior to the wave collision area (while considering the other aspects of APLE mapping). This area is marked on the 3D map. As the wave collision often occurs over a broad area, the wave collision is demonstrated by the black outline in **Figure 2**.

#### Low-voltage mapping

**Figure 3:**
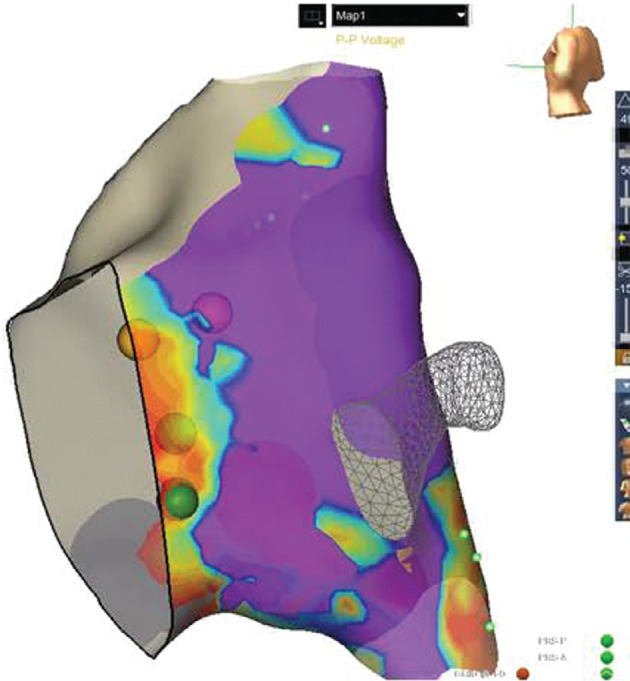
An example of the voltage map for patient 1. The purple color represents the higher-voltage atrial signals of ≥1.5 mV, and the red/orange areas represent the low voltages near 0.1 mV. The gradient between red and purple represents increasing voltages. The His bundle is indicated by the gold spheres, and two of the cryolesions are represented by the large green spheres overlying the low-voltage area.

The lowest setting of the voltage gradient bar was initially set at 0.1 mV, with the upper voltage typically set between 1.5–2 mV. This can be adjusted slightly to highlight the low-voltage areas (down to 0.05 mV). Areas of low voltage near 0.1 mV (orange) were considered areas of interest for potential slow-pathway ablation when accounting for other components of APLE mapping **(Figure 3)**.

#### Atrial signal appearance

After evaluating the potential slow-pathway ablation sites based on the three prior techniques, areas of interest were assessed for long-duration (>40 ms) atrial signals. As these had previously been evaluated for low voltage, these typically showed the typical low-voltage long-duration signal appearance.

#### Final selection of the ablation site

Areas of interest that combined later activation, at or superior to the wave collision, at a site with low voltage and with long-duration atrial electrograms were selected for possible ablation.

#### Ablation

If sustained AVNRT was possible, patients were placed in the tachycardia prior to ablation. Uniformly, a 6-mm–tip Freezor Xtra 3 catheter was used for the ablation (Medtronic, Minneapolis, MN, USA). Applications of cryotherapy were given during AVNRT at a previously selected site (based on APLE mapping discussed already) while using a trigger screen off an atrial signal to monitor both the AV nodal conduction time and the beat-to-beat interval. This allowed close monitoring for alterations in either the slow pathway or AV nodal conduction. If there was no effect within 20 s, the cryolesion was stopped. However, if there was slowing and termination of the AVNRT (to sinus) within 20–30 s, then a 5-min application was delivered, followed by a freeze–thaw–freeze cycle at the successful site. Based on our institutional practice, at least four additional 4-min applications were placed around the successful site.

If there were multiple areas that qualified as potential ablation sites using the APLE technique, the initial site of ablation was selected to be more distant from the AV node for safety, even if it was less attractive for first lesion success.

After ablation, post-testing to evaluate for recurrence of slow-pathway conduction or SVT was repeated on and off isoproterenol for ≥30 min before catheter removal. As is our institutional preference, if single echo beats remained present, even if there were no inducible arrhythmias, additional cryo applications were placed until either eradication of the echo beats or evidence of any (reversible) unwanted AV nodal effect.

After the procedure, patients completed two follow-up visits, typically at 6–8 weeks and then again at 1 year.

## Results

Five patients who underwent mapping of the slow pathway using the HD Grid at our institution were retrospectively evaluated for this study. The median age was 14 years (12–15 years), the median weight was 54.1 kg (46–77 kg), and the median height was 161.5 cm (151–180 cm). Mapping characteristics are shown in **Table 1**.

After confirmation of AVNRT, the TOK was mapped for a median of 3 min (2:30–4:37 min). Overall, a median of 154 data points (89–217 data points) were acquired on the surface of the TOK; data points not directly on the surface of the TOK were not counted. Ablation characteristics are shown in **Table 2**.

### Ablation sites of success

**Figure 4:**
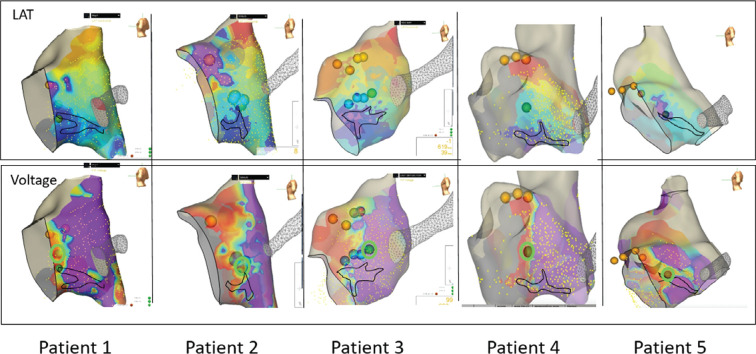
Top row: local activation time mapping for each patient with the wave collision marked in black. The local activation time shows progression of the atrial conduction during sinus rhythm with the earliest signal in red and the latest signal in purple. Selected cryolesions are also displayed. Bottom row: Voltage mapping for each patient, again with the location of the wave collision marked. The voltage map has the lowest-voltage signals set at 0.1 mV (red/orange) and the higher voltages (purple) above 1.5–2 mV. The successful cryolesion is highlighted in green on the voltage map. Ablation site selection used the activation, propagation, low-voltage, and electrogram technique (as discussed already) to determine the site of initial ablation attempts. *Abbreviation:* LAT, local activation time.

**Figure 4** demonstrates the LAT map of the TOK from a left posterior lateral view with the site of wave collision marked (top row) and the voltage map included (bottom row). The site of the first successful lesion is highlighted in green on the voltage map. The site of wave collision is outlined in black on maps to allow an understanding of the location of wave collision in comparison to the low-voltage areas. Notice that there was generally late activation, and the wave collision was near the mid- to low TOK. Not all cryolesions are included in the figure to facilitate clarity of the display of localization of the successful site.

Ablation was attempted during SVT in all but one patient (patient 2—who had only non-sustained SVT during the ablation portion of the procedure). The median number of lesions to success was 3 (range was 1–15 lesions to success). Even so, the two patients with success after lesion 3 included patient 1, who had long-term success at lesion 5, but also had slowing and termination of the arrhythmia on lesion 2 (which was not considered the successful lesion). After finishing lesion 2, a slower AVNRT was induced and ablated on the fifth lesion, which was considered the successful lesion. A similar effect occurred with patient 3 on the first application, but with continued echo beats and recurrence of a slower non-sustained AVNRT, which were eventually eliminated with cryo application 15. Additionally, our practice is, if there are multiple sites, to choose the site felt to be the safest even if we feel that it will less likely result in success on the first application.

The median number of total cryolesions was 11 (range, 6–19), with a median duration of therapy of 30 min and 33 s (25–36 min). Our practice is, at the site of success, to place an additional lesion at the same site (a freeze–thaw–freeze cycle) and then place the surrounding lesions overlapping the edges of the successful site (for a minimum of six total lesions). If an AH “jump” from the fast to slow pathway or an echo beat was noted, then additional lesions were typically placed in an attempt to eliminate the jump or echo.

The location of the successful lesions is shown in **Figure 4** (bottom row) and highlighted in green. In all but one patient, the successful site was overlying the lowest-voltage areas. The patient without the successful lesion overlying the lowest-voltage area was patient 3, and, as discussed already, this patient had transient success on the first ablation over low-voltage areas at a site just superior to the wave collision over an area of late activation with long-duration electrograms. However, this patient experienced recurrence with a slower AVNRT and an eventual elimination of the slow pathway at the border of a lower- and higher-voltage area on lesion 15, circled in green.

Patient 1 continued to have a single echo beat despite additional short-duration cryotherapy attempts to eliminate the single echo beats. Patient 5 continued to have a “jump” of ∼50 ms with a 10-s decrement in extrastimulus, but no other evidence of slow-pathway conduction. In all patients, the A–A time was greater than the A–V time at Wenckebach post-ablation, a change from pre-ablation in all patients. There was no inducible SVT at the end of any procedure, and there have been no recurrences during follow-up of 1 year in all patients. There were no complications. No radiation was used.

## Discussion

We appreciate that, despite years of experience in the treatment of AVNRT, there continue to be both recurrences and complications. The recurrence risk for AVNRT can be as high as 5%–10%, and the risk of heart block (with radiofrequency [RF]) is approximately 1%.^2,3^ More accurate localization of the slow pathway within the TOK would be expected to both improve success and decrease complications. This combination of a lack of success and a lack of accuracy suggests that further improvements in AVNRT ablation technique remain necessary.

This is the first study to evaluate the use of the HD Grid for high-density electrogram mapping using the APLE technique. As discussed already, the APLE technique integrates late activation, propagation wave assessment (wave collision), low-voltage mapping, and the appearance of the electrogram. This technique (without the HD Grid) has been performed at our institution for years and has had a high success rate. However, we were interested in the ability of the HD Grid to increase the electrogram point density and potentially improve the accuracy of mapping the slow pathway.

The APLE technique was successful in all five patients. The median number of lesions to success was 3, but the two patients above the median both had an alteration of the tachycardia within the first two ablations, suggesting that the re-entrant circuit was partially disrupted despite a greater number of lesions to long-term success. If there were multiple potential sites considered for ablation, our institutional practice was to first choose the site further from the His bundle, even if this seems less promising for immediate success. Therefore, we feel that a median success at lesion 3 reinforces the value of this technique and, at least until this or other electrically guided techniques are more extensively validated, the philosophy of choosing a safer site is appropriate. This is particularly important if RF is chosen.

The median total number of applications was 11. Again, our institutional practice is to place additional lesions at and around the successful site, reaching a minimum of six lesions with initial success. We then attempt to ablate single echo beats. We appreciate that this small study does not evaluate the technique for superiority over other methods and that it cannot be compared to RF ablation, but we also feel that it adequately demonstrates the potential for APLE technique success with high-density mapping.

Without the HD Grid, our institution generally seeks an atrial signal point density in the TOK of ≥35–40 points. In comparison, we quickly acquired a median of 4× greater point density in a median of 3 min. This is relatively quick, but, at the same time, it does not account for the time to acquire, heparinize, and place the HD Grid.

In comparison to lower-point-density, single-catheter mapping, we did feel that the higher point density allowed the LAT and propagation wave to be more distinguished, especially when evaluating the direction and speed of the wavefront. Unfortunately, this study was completed shortly before we obtained the updated version of the mapping system, so we were unable to assess the impact of omni-directional wavefront analysis. When not using the HD Grid, our institution obtains atrial signal points during geometry creation with the steerable catheter (even before confirmation of the rhythm type), limiting the time to map with a standard catheter. Therefore, though mapping with the HD Grid is rapid and has a substantially greater point density, we do not feel it provides a substantial time advantage for mapping the TOK over using a standard catheter. Similar to a paper by Drago et al., subjectively, we found that the voltage maps using the HD Grid generally also demonstrated a higher voltage than that achieved when using a steerable decapolar catheter.^9^ We expect that this is due to the ability of the HD Grid to display the peak amplitude of wavefronts that are perpendicular to the bipole of a standard catheter traversing the TOK. Given the likely inferior-to-superior direction of the slow-pathway conduction, multi-directional mapping may offer a long-term advantage for pathway localization.

Though mapping was performed during sinus rhythm, ablation was performed during SVT. We appreciate that alterations in heart rate can occasionally shift the map slightly; however, we did not find this to be a significant issue in these patients. We chose to ablate during SVT to allow for very quick and accurate assessment of the location and effect on the slow pathway. We use short-duration test applications (≤20–30 s) and, if there is no slowing and termination within that time, creation of the lesion is stopped and a new site is chosen. Unlike RF energy that “paints” the surrounding tissue due to cardiac movement, affixing to the tissue allows excellent accuracy to the site of success and consequently helps to localize the slow pathway. This rapid assessment of a slow-pathway effect is not possible with an ablation in sinus rhythm. In the future, we may consider mapping of the arrhythmia during SVT; however, we currently feel that the challenges outweigh the benefits. In particular, we are concerned about the interpretation of late low-voltage atrial signals with overlying far-field ventricular signals, the lack of patient tolerance to SVT, the potential for a lack of sustained arrhythmia, and the desire to have a technique that is easily reproducible without requiring tachycardia. If, with further study, we find that the APLE technique and/or other electrically guided techniques mapped during sinus rhythm are not adequate, these concerns may need to be addressed.

Lastly, with higher point density, all five of our patients experienced a change in the direction of the atrial signal toward the His bundle after it passed inferiorly through the mid-TOK, as demonstrated in **Figure 1**. Bailin (who originally described the low-voltage AVNRT technique) termed the change in direction of the wavefront to be the pivot point, which they felt correlated with the site of successful ablation.^10^ In our experience, late activation, near the ventricular side of the TOK, more ventricular and superior than this pivot point, appears to correlate with successful ablation sites using APLE mapping. We expect this pivot point may represent the “entrance” to the slow pathway given that this territory demonstrates the substrate required for a re-entrant circuit. In particular, there is unidirectional block within the superior TOK (often along the His/tendon of Todaro area), a limb back to the His-bundle area (as noted with the change in direction), and slow conduction to allow fast pathway repolarization. We will need to consider if this pivot point or other factors, such as duration, directionality, and/or velocity, are more accurate localizers than the wavefront collision with further studies.^11^ Our successful sites were often on the ventricular side of the TOK; we appreciate that this is more ventricular than often expected using the anatomic approach. We expect that ablation closer to the CS (especially with RF) may ablate a somewhat broader entrance to the slow pathway and sites more ventricular may ablate more distal in the slow pathway, both potentially achieving successful AVNRT slow-pathway ablation.

As with any small study, we appreciate the limitations. Our experience with this technique is without comparison to the current techniques, limiting any conclusions gained from a head-to-head comparison. We also appreciate the added cost and need for clinical validation of this technique with the use of the HD Grid. Even so, we appreciate the potential patient and hospital benefit if this or similar techniques can decrease or eliminate the risk of complications and/or the risk of recurrence.

## Conclusion

This case series suggests that mapping with the HD Grid using the APLE mapping technique can be successful, results in high-density mapping points, and is relatively quick. Despite consistent success in this small case series, we also appreciate the need for further investigation regarding the APLE technique with high-voltage mapping and appreciate the consideration of cost, anticoagulation, and the implications for evolving ablation techniques.

## Figures and Tables

**Video 1:** The propagation map used to evaluate and mark the site of the wave collision. The white wave represents the front edge of electrical conduction through the triangle of Koch. The yellow lesions represent the His signals and the green applications were areas of successful lesions, with the inferior lesion slowing the atrioventricular nodal re-entry tachycardia and the superior lesion terminating the atrioventricular re-entrant tachycardia.

**Video 2:** A sparkle map also representing the duration of atrial signals. This map was not used for determination of the site to ablate in the present study, but it is a useful alternative means of evaluating the atrial signals within the triangle of Koch.

**Table 1:** Mapping characteristics

	Time to Map TOK (min:s)	Data Points on the Geometry Surface Within the TOK
Median	3:00	154
Range	2:30–4:37	89–217

**Table 2:** Ablation characteristics

	No. of Cryolesions to Success	Total Number of Lesions	Duration of Therapy (min:s)
Patient 1	5^a^	11	33:26
Patient 2	2	8	26:11
Patient 3	15^a^	19	36:27
Patient 4	3	15	30:33
Patient 5	1	6	25:00
Median	3	11	30:33

^a^Patients 1 and 5 both had slowing of the AVNRT on either the first or second applications, but a slower AVNRT was induced during post-ablation testing, so the initial lesions were not considered successful.

## References

[1] Orejarena LA, Vidaillet H, DeStefano F (1998). Paroxysmal supraventricular tachycardia in the general population. J Am Coll Cardiol.

[2] von Bergen NH, Law IH (2012). AV nodal reentrant tachycardia in children: current approaches to management. Prog Pediatr Cardiol.

[3] Makker P, Saleh M, Vaishnav AS (2021). Clinical predictors of heart block during atrioventricular nodal reentrant tachycardia ablation: a multicenter 18-year experience. J Cardiovasc Electrophysiol.

[4] Bailin SJ, Korthas MA, Weers NJ, Hoffman CJ (2011). Direct visualization of the slow pathway using voltage gradient mapping: a novel approach for successful ablation of atrioventricular nodal reentry tachycardia. Europace.

[5] Malloy L, Law IH, von Bergen NH (2014). Voltage mapping for slow-pathway visualization and ablation of atrioventricular nodal reentry tachycardia in pediatric and young adult patients. Pediatr Cardiol.

[6] Marshall AM, Erickson CC, Danford DA, Kugler JD, Thomas VC (2019). The low specificity of low voltage bridges associating atrioventricular nodal reentry in pediatric patients. J Interv Card Electrophysiol.

[7] van Aartsen A, Law IH, Maldonado JR, Von Bergen NH (2017). Propagation mapping wave collision correlates to the site of successful ablation during voltage mapping in atrioventricular nodal reentry tachycardia. J Innov Card Rhythm Manag.

[8] Drago F, Calvieri C, Allegretti G, Silvetti MS (2020). Mapping of low-voltage bridges with a high-density multipolar catheter in a child with atrioventricular nodal reentry tachycardia. HeartRhythm Case Rep.

[9] Drago F, Tamborrino PP, Porco L (2022). Koch’s triangle voltage mapping for cryoablation of slow pathway in children: preliminary data of a novel high-density technique. J Interv Card Electrophysiol.

[10] Bailin SJ, Rhodes TE, Arter JC, Kocherla C, Kaushik N (2022). Physiology of slow pathway conduction during sinus rhythm: evidence from high density mapping within the triangle of Koch. J Interv Card Electrophysiol.

[11] Howard TS, Valdes SO, Zobeck MC (2022). Ripple mapping: a precise tool for atrioventricular nodal reentrant tachycardia ablation. J Cardiovasc Electrophysiol.

